# Changes in the pharyngeal and nasal microbiota in pediatric patients with adenotonsillar hypertrophy

**DOI:** 10.1128/spectrum.00728-24

**Published:** 2024-09-09

**Authors:** Federica Del Chierico, Antonia Piazzesi, Ersilia Vita Fiscarelli, Maria Vittoria Ristori, Ilaria Pirona, Alessandra Russo, Nicoletta Citerà, Gabriele Macari, Sara Santarsiero, Fabrizio Bianco, Valeria Antenucci, Valerio Damiani, Luigi Mercuri, Giovanni Carlo De Vincentis, Lorenza Putignani

**Affiliations:** 1 Research Unit of Microbiome, Bambino Gesù Children’s Hospital, IRCCS, Rome, Italy; 2 Research Unit of Diagnostical and Management Innovations, Bambino Gesù Children’s Hospital, IRCCS, Rome, Italy; 3 GenomeUp SRL, Rome, Italy; 4 Unit of Microbiomics, Bambino Gesù Children’s Hospital, IRCCS, Rome, Italy; 5 Unit of Otorhinolaryngology, Bambino Gesù Children’s Hospital, IRCCS, Rome, Italy; 6 Quality Team Studi Clinici, Bambino Gesù Children’s Hospital, IRCCS, Rome, Italy; 7 Modelli Innovativi di Regolamentazione in Pediatria, Bambino Gesù Children’s Hospital, IRCCS, Rome, Italy; 8 Medical Department, DMG Italia SRL, Pomezia, Italy; 9 Unit of Microbiomics and Research Unit of Microbiome, IRCCS, Bambino Gesù Children’s Hospital, Rome, Italy; University of Washington, Seattle, Washington, USA

**Keywords:** adenotonsillar hypertrophy, pharyngeal microbiota, nasal microbiota, upper airway pathobionts, probiotics

## Abstract

The present study aimed to investigate the pharyngeal and nasal microbiota composition in children with adenotonsillar hypertrophy (AH) and assess longitudinal alterations in both microbiota after a probiotic oral spray treatment. A cohort of 57 AH patients were enrolled and randomly assigned to the probiotic and placebo groups for a 5-month treatment course. Pharyngeal and nasal swabs were collected before and after treatment and analyzed by 16S rRNA-based metataxonomics and axenic cultures for pathobiont identification. 16S rRNA sequences from pharyngeal and nasal swabs of 65 healthy children (HC) were used as microbiota reference profiles. We found that the pharyngeal and nasal microbiota of AH children were similar. When compared to HC, we observed an increase of the genera *Rothia*, *Granulicatella*, *Streptococcus*, *Neisseria*, and *Haemophilus*, as well as a reduction of *Corynebacterium*, *Pseudomonas, Acinetobacter,* and *Moraxella* in both microbiota of AH patients. After probiotic treatment, we confirmed the absence of adverse effects and a reduction of upper respiratory tract infections (URTI). Moreover, the composition of pharyngeal microbiota was positively influenced by the reduction of potential pathobionts, like *Haemophilus* spp., with an increase of beneficial microbial metabolic pathways. Finally, the probiotic reduced the abundance of the pathobionts *Streptococcus mitis* and *Gemella haemolysans* in relation to AH severity. In conclusion, our results highlight the alterations of the pharyngeal and nasal microbiota associated with AH. Moreover, probiotic administration conferred protection against URTI and reduced the presence of potential pathobionts in patients with AH.

**IMPORTANCE:**

Adenotonsillar hypertrophy (AH) is considered the main cause of breathing disorders during sleep in children. AH patients, after significant morbidity and often multiple courses of antibiotics, often proceed to tonsillectomy and/or adenoidectomy. Given the potential risks associated with these procedures, there is a growing interest in the use of nonsurgical adjuvant therapies, such as probiotics, that could potentially reduce their need for surgical intervention. In this study, we investigated the pharyngeal and nasal microbiota in patients with AH compared with healthy children. Furthermore, we tested the effects of probiotic spray administration on both disease symptoms and microbiota profiles, to evaluate the possible use of this microbial therapy as an adjuvant for AH patients.

## INTRODUCTION

Adenotonsillar hypertrophy (AH) is considered the main cause of breathing disorders during sleep in children, ranging from snoring to obstructive sleep apnea syndrome, which, in turn, can cause cardiovascular disease, metabolic disorders, behavioral disorders, and learning delays (1, 2). The adenoids and tonsils are, respectively, part of the nasal- and mucosa-associated lymphoid tissue systems, which are the host’s first site of contact with respiratory antigens (3). Though the etiopathogenesis of AH in children is unknown, it is assumed that bacteria, host genetics, and epigenetic factors can precipitate a detrimental immune response (4). Moreover, as the surface of the adenoids and tonsils are colonized by commensal microorganisms (5), cross-talk between the host’s immune system and the upper airway microbiota could be important in either protecting or predisposing the host to mucosal infection and AH (6). AH patients, after significant morbidity and multiple courses of antibiotics, are often required to proceed with tonsillectomy and/or adenoidectomy (7). Since these surgical procedures present a potential risk of adverse events (7), there is a growing need for the development of nonsurgical therapeutic alternatives, such as probiotics, for these patients (8, 9).

To address this need, we characterized the adenotonsillar microbiota in patients affected by AH compared to healthy children (HC), to see whether, indeed, AH is associated with alterations in the upper respiratory microbial environment. Moreover, we evaluated the effects of an oral spray probiotic, both on the clinical manifestations of this disease and on adenotonsillar microbial ecology.

## MATERIALS AND METHODS

### Study design

**Fig 1:**
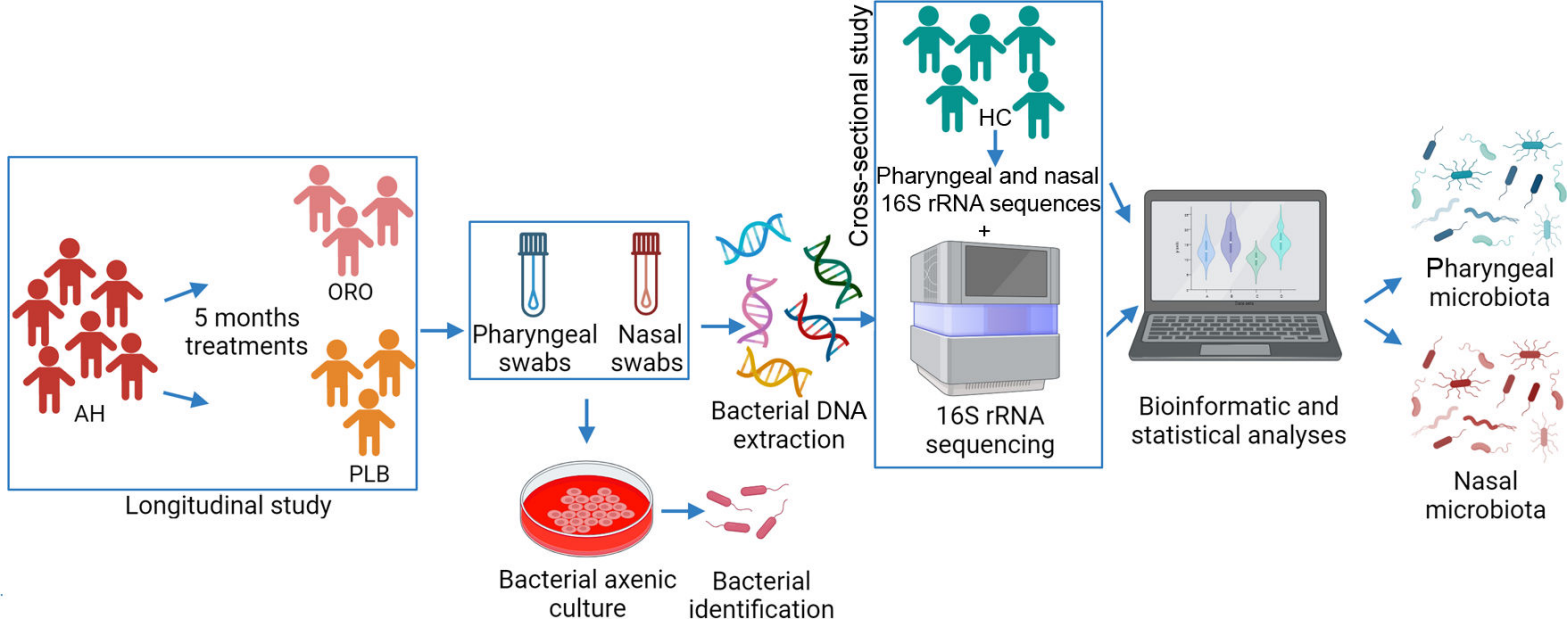
Study workflow.

This study was made up of two parts. The first part was a cross-sectional study, which compared the profiles of the pharyngeal and nasal microbiota in AH children and HC. The second part was a longitudinal study, which investigated the effects of the administration of an oral spray probiotic on AH clinical symptoms and pharyngeal and nasal microbiota composition (Fig. 1).

### Study group

Children affected by grade II–IV AH with inflammation, in consultation for tonsillectomy, were enrolled at the Department of Otorhinolaryngology of the Bambino Gesù Children’s Hospital, IRCCS, at Rome, Italy, between March 2019 and February 2021. The inclusion criteria were grade II–IV AH with inflammation with persisting symptoms for at least 1 year, ages 3–8 years, and enrolment in the surgical wait list for tonsillectomy for recurrent inflammation (10). The exclusion criteria were rheumatic and autoimmune diseases, craniofacial malformation syndromes, neuromuscular diseases, the presence of both suppurative and nonsuppurative manifestations, severe asthma, and obesity with body mass index (BMI) ≥40. Moreover, patients who received antibiotic treatment up to 15 days before the enrolment and/or steroid treatment up to 1 month before the enrolment or those who did not provide informed consent for the study were excluded. Adenoid and tonsillar hypertrophy was confirmed by pharyngoscopy and classified into 4 grades of severity based on adenoid and tonsillar size. Grades of tonsil hypertrophy have been previously reported (11). Briefly, grade 1 (G1) tonsils were in the tonsillar fossa, barely visible behind the anterior pillars; grade 2 (G2) tonsils were easily visible behind the anterior pillars; grade 3 (G3) tonsils extended three-fourths of the way to the midline; and grade 4 (G4) tonsils were completely obstructing the airway.

Adenoid hypertrophy was graded according to Parikh’s classification based on the anatomical relationships between the adenoid tissue and the surrounding structures: vomer, soft palate, and torus tubarius. Grade 1 adenoids are nonobstructive and do not contact any of the previously mentioned anatomic subsites, while grade 2, 3, and 4 adenoids contact the torus tubarius, vomer, and soft palate, respectively (12). Tympanometry was performed to evaluate the middle ear’s ventilation state.

### Intervention

After baseline examination, the patients were randomly (1:1) treated with probiotic oral spray (ORO) or hypertonic oral spray (PLB). The probiotic spray consisted of a minimum of 125 × 10^9^ colony-forming units (CFU)/g of lyophilized *Streptococcus salivarius* 24SMBc and *Streptococcus oralis* 89a. The placebo spray consisted of the same excipients of the probiotic spray polyethylene glycol (PEG) / polypropylene glycol (PPG) copolymer, fructose, PEG-14 dimethicone, buffered isotonic solution at pH 5.5, and aroma) without the “active” components (13). Both were administered daily, two sprays per administration, for 5 consecutive months (14).

Each patient underwent three clinical evaluations by an otorhinolaryngology specialist: visit 1, a month after randomization; visit 2, after 3 months of randomization; and, finally, visit 3, after 5 months of treatment. During each visit, the following parameters were evaluated and recorded: complete physical examination, body temperature, signs of chronic or acute adenotonsillar inflammation, drug consumption, and adverse events.

### 16S rRNA sequencing of oral microbiota

Two hundred twenty-eight biological samples were collected from patients at *T*_0_ (before treatment) and *T*_1_ (after 5 months of treatment) and either processed immediately or stored at −80°C until analysis. DNA was automatically extracted using the EZ1 DNA Tissue Kit and biorobot EZ1 extractor following the manufacturer’s instructions (Qiagen, Hilden, Germany). The V3-V4 region of the 16S rRNA gene (~460 bp) was amplified by PCR using the primers 16S_F 5′-(TCG TCG GCA GCG TCA GAT GTG TAT AAG AGA CAG CCT ACG GGN GGC WGC AG)-3′ and 16S_R 5′-(GTC TCG TGG GCT CGG AGA TGT GTA TAA GAG ACA GGA CTA CHV GGG TAT CTA ATC C)-3′ as reported in the MiSeq rRNA Amplicon Sequencing protocol (Illumina, San Diego, CA). The first amplification was carried out under the following conditions: initial denaturation at 95°C for 3 min, 32 cycles of denaturation at 95°C for 30 s, annealing at 55°C for 30 s, extension at 72°C for 30 s, and final extension step at 72°C for 5 min, with the Fast Start Hifi Taq kit (Roche Diagnostics, Mannheim, Germany). DNA amplicons were purified with 20 µL of KAPA Pure Beads (Roche Diagnostics, Mannheim, Germany) and tagged with unique index combinations using Nextera technology in a second amplification step. The following steps consisted of library purification, quantification with Quant-iT PicoGreen dsDNA Assay Kit (Thermo Fisher Scientific, Waltham, MA), and dilution to 4 nM. Pooling, denaturation, and dilution to 7 pM were performed before sequencing on an Illumina MiSeq platform (Illumina, San Diego, CA, United States) where paired-end reads of 300 base lengths were generated. Sequencing data files and metadata from HC were downloaded from a public repository (PRJNA554533) as indicated in the original publication (15).

### Biocomputational and statistical analyses

Paired-end sequencing reads in fastq format were analyzed using QIIME2 (v2023.2) (16). The QIIME2 plugin for DADA2 was used for quality control, denoising, chimera removal, trimming, and construction of the amplicon sequence variant (ASV) table (17). Taxonomic analysis was performed using a Naive Bayes model pre-trained on Greengenes2 2022.10 through the QIIME2 plugin q2-feature classifiers (18). Before proceeding with α- and β-diversity analyses, removal of unassigned ASVs and rarefaction of samples was performed. All ecological statistical analyses were performed using the microeco R package (R v4.3.0) (19). Statistical analyses on α-diversity indices were performed using the nonparametric Mann–Whitney and Kruskal–Wallis tests. The PERMANOVA test was applied to β-diversity matrices. For further analyses, a raw feature table was normalized with the cumulative sum scaling method (20). For high-dimensional biomarker discovery and explanation, an algorithm based on linear discriminant analysis (LDA) effect size (LEfSe) was used (21). Seasonality was evaluated as a confounding factor with the microbiomeMarker v1.6.0 R package and the Microbiome Multivariable Association with Linear Model 2 (MaAsLin2) algorithm (22).

Phylogenetic Investigation of Communities by Reconstruction of Unobserved States (PICRUSt) exploiting the Kyoto Encyclopedia of Genes and Genomes orthologs (KO) database was used to determine ASVs and their functional potential (23). Network analysis was conducted using the library in R Netcomi as previously described (24). To investigate the bacterial network characteristics of each ecological niche, the 50 most highly expressed bacterial genera in each group were selected, on which Pearson’s correlation and relative abundance normalization, based on the centered log ratio, were applied. Cut-offs of rho ≥0.6 and false discovery rate (FDR) corrected *P*-value ≤0.05 were applied. The differential networks were obtained by selecting the 80 most highly expressed bacterial genera. Fisher’s *z*-test and multiple testing adjustment, performed via FDR, were used to select the genera that are differentially associated among the sample groups.

### Pathobiont detection by microbiological culture-based methods

The pathobionts *Enterobacter cloacae*, *Gemella haemolysans*, *Haemophilus influenzae*, *Haemophilus parainfluenzae*, *Neisseria flavescens*, *Neisseria subflava*, *Rothia mucilaginosa*, *Staphylococcus aureus*, *Streptococcus mitis*, *Streptococcus oralis*, *Streptococcus parasanguis*, *Streptococcus pyogenes*, *Streptococcus salivarius*, and *Streptococcus vestibularis* were selected for microbiological culture.

Swabs from patients were vortexed in 10 µL of liquid medium and plated at 37°C for 48 h in the following media:

Aerobes: Columbia agar with 5% sheep blood, MacConkey agar, Mannitol salt agar, Pseudosel agar, and Tellurite Blood agar
Anaerobes: Schaedler K agar with 5% sheep blood with or without kanamycin and vancomycin and Bacteroides bile esculin agar with amikacin, which were incubated in the presence of 85% N_2_, 10% H_2_, and 5% CO_2_
Facultative anaerobes: Columbia nalidixic acid agar with 5% sheep blood with an optochin disk, Chocolate agar with and without bacitracin, Bourdet–Gengou agar, Bordetella selective agar, and Modified Thayer–Martin agar, which were incubated in the presence of 5%–8% CO_2_

All colony morphotypes observed on the selective and nonselective media were identified by proteomic profiling by matrix-assisted laser desorption-time of flight mass spectrometry (Bruker Daltonics, Bremen, Germany).

### Statistical analysis

IBM SPSS Statistics 21 software (IBM Corp., New York, United States) was used for statistical analysis of the anthropometric and clinical data. The Shapiro–Wilk test and Kolmogorov–Smirnov test were used to test data distribution. The *t*-test was used on normally distributed data. The Wilcoxon signed-rank test was used to compare related samples. The Mann–Whitney *U* test was used to compare two independent samples, while the Kruskal–Wallis *H* test was used to compare three or more independent samples. Chi-squared tests or Fisher’s exact test was used for categorical variables.

## RESULTS

### Description of participants

**Fig 2:**
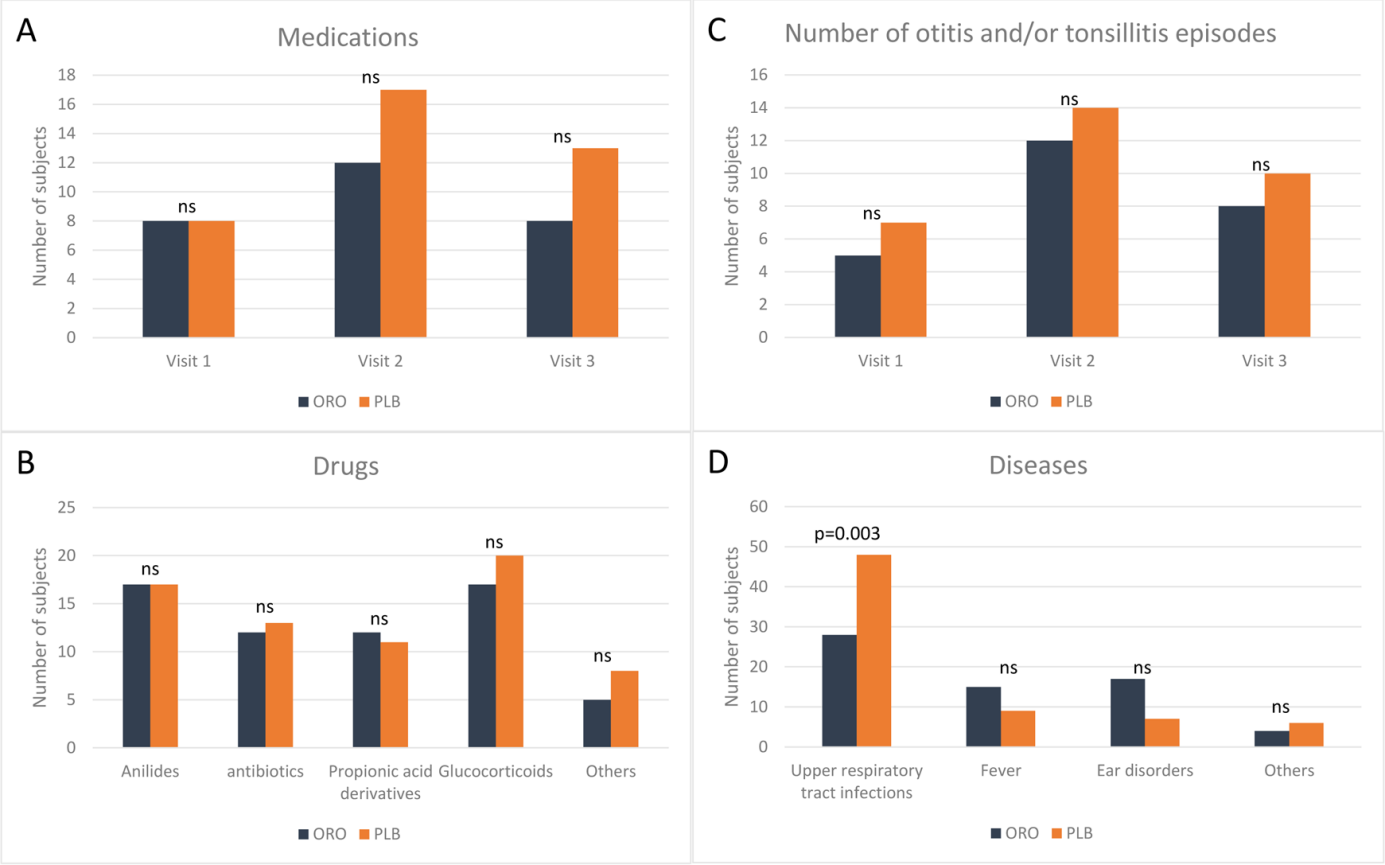
Histograms of clinical features of AH cohorts. (**A**) Medications administered during the entire follow-up time. (**B**) Drugs administered during the entire follow-up time. (**C**) Number of otitis and tonsillitis episodes registered during the entire follow-up time. (**D**) Diseases registered during the follow-up period.

We compared the demographic data of 57 AH children and 65 HC. No significant differences in age or gender were observed between the two groups (Table 1). In the longitudinal study, the 57 AH patients were randomly assigned to receive either the probiotic spray (ORO group, 27 patients) or the placebo spray (PLB group, 30 patients) with two sprays daily for 5 months (Table 1). *T*_0_ was defined as the day before treatment began, and *T*_1_ was after the full 5-month course of treatment was completed. Data on medication, drug administration, diseases, and number of otitis and/or tonsillitis episodes between the ORO and PLB groups were also collected (Fig. 2). No statistical differences were found between the two groups, except for the number of upper respiratory tract infections (URTI) that were increased in the PLB group (Fig. 2A through D).

**TABLE 1:** Study population characteristics^*c*^

Features	AH						HC	*P*-values
Subject number	57						63	
Age (years) average ±SD	5.6 ± 1.4						5.5 ± 0.9	0.26^*a*^
Gender	23F/34M						34F/29M	0.28^*b*^

	ORO			PLB				*P*-values
Subject number	27			30				
Age (years) (average ±SD)	5.5 ± 1.4			5.6 ± 1.4				0.76^*a*^
Gender	14F/13M			9F/21M				0.28^*b*^
BMI (kg/m^2^) (average ±SD)	17.11 ± 3.09			15.52 ± 1.72				0.02^*a*^
Drugs								
Anilides	17			17				0.46^*b*^
Antibiotics	12			13				0.60^*b*^
Propionic acid derivatives	12			11				0.76^*b*^
Glucocorticoids	17			20				0.48^*b*^
Others	5			8				0.34^*b*^
Diseases								
Upper respiratory tract infections	28			48				0.00^*b*^
Fever	15			9				0.97^*b*^
Ear disorders	17			7				1.00^*b*^
Others	4			6				0.43^*b*^
Grading classes/*N* patients	G1	G2	G3	G1	G2	G3		
*T*_0_	0	15	12	0	20	10		0.27^*b*^
*T*_1_	3	17	7	6	19	5		0.18^*b*^

^*a*^ *t*-test *P*-values.

^*b*^ Fisher’s exact test *P*-values.

^*c*^ Demographic characteristics for AH and HC children are reported. Clinical and instrumental features are reported for the AH group stratified for probiotic (ORO) and placebo (PLB) treatment, before (*T*_0_) and 5 months after (*T*_1_).

### Dysbiosis of the pharyngeal and nasal microbiota in AH

A total of 886,512 sequencing reads were obtained with a sampling depth of 3,036.00 and a mean value of 77,113.73 sequences per sample. We identified a total of 18,629 ASVs, grouped in 20 phyla and 270 bacterial families (Table S1).

First, we evaluated the influence of seasonality on the microbiota composition of the nose and pharynx. We confirmed that seasonality did not have a significant effect on the bacterial composition of the nasal microbiota, while it did affect the pharyngeal one (Table S2). We then identified which microbes were affected by the seasons in the pharynx, including *Akkermansia, Klebsiella*_724518, *Dialister, Neobacillus, Mannheimia, Clostridium*_AP, and *Eisenbergiella*, which increased during autumn; *Bulleidia* and *Catonella*, which increased in the spring; and *Moraxella*_C_651924, *Avispirillum,* and *Aphodomorpha*, which increased during winter (Table S3; Fig. S1).

**Fig 3:**
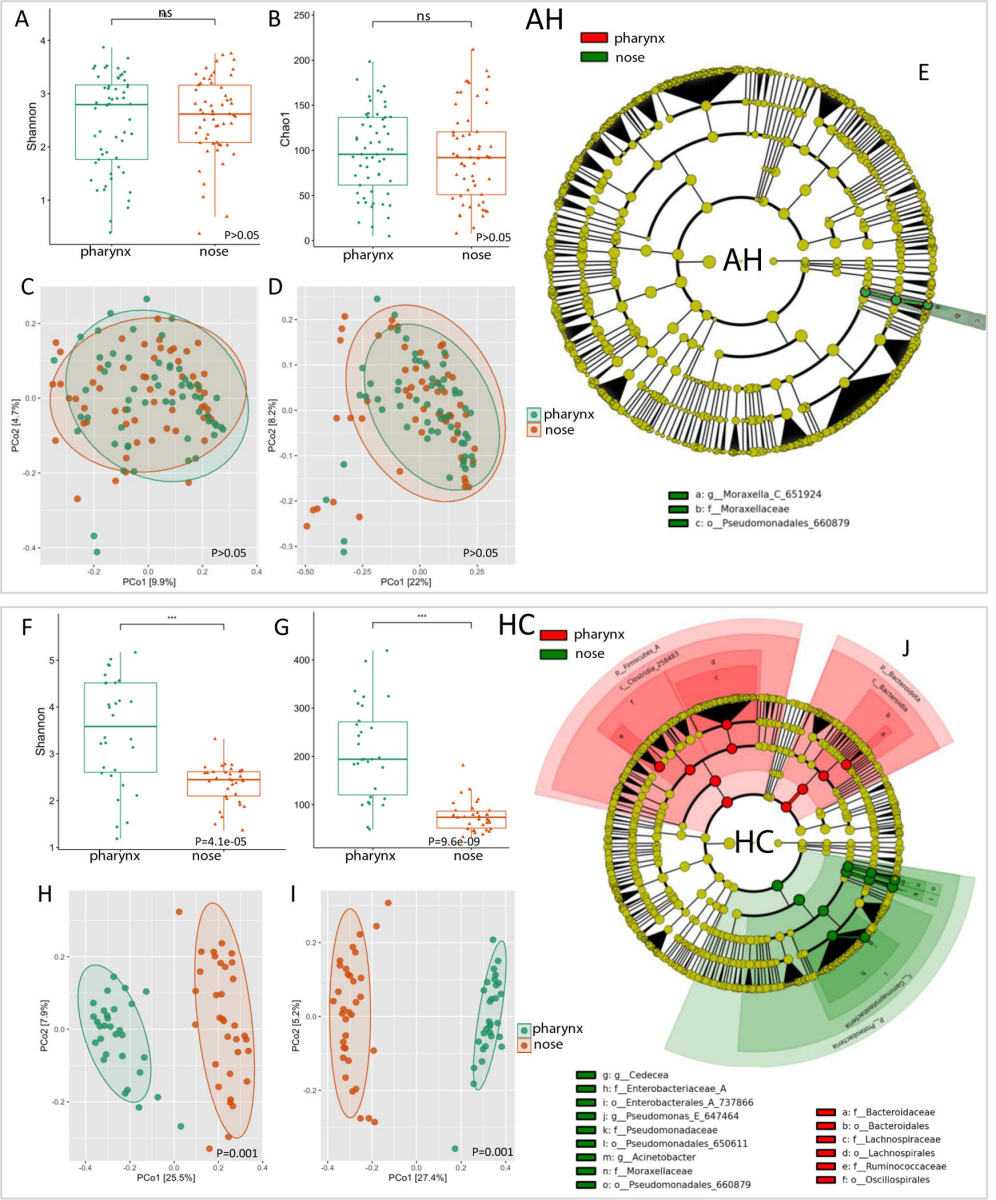
Pharyngeal and nasal microbial ecosystems in AH and HC. Alpha-diversity box plots of pharyngeal and nasal microbiota based on Shannon and Chao1 indexes (**A and B, F and G**). *P*-values obtained by the Mann–Whitney test are reported. Beta-diversity Principal Coordinates Analysis (PCoA) plots of pharyngeal and nasal microbiota, performed by Bray–Curtis and unweighted UniFrac algorithms (**C and D, H and I**). *P*-values obtained by the PERMANOVA test are reported. LEfSe cladograms of pharyngeal and nasal samples (**E and J**). Taxa and nodes highlighted in green and red were significantly more abundant in AH patients and HC, respectively.

To describe the differences between the pharyngeal and nasal microbial communities, we measured ecological parameters based on α-diversity (Shannon and Chao-1 indexes) in AH and HC (Fig. 3A, B, F, and G). The ratio between pharyngeal and nasal microbial richness was significantly decreased in AH (1.120) with respect to HC (1.525) (*P*-value =0.0148). The small differences between the pharyngeal and nasal microbial ecosystems in AH were also confirmed by β-diversity analysis. Conversely, the differences between the pharyngeal and nasal microbiota in HC were more pronounced (Fig. 3C, D, H, and I). LEfSe analysis showed an increase of the genus *Moraxella* in nasal swabs of AH patients compared to pharyngeal ones (Fig. 3E). In HC subjects, on the other hand, the *Moraxella*, *Pseudomonas*, *Acinetobacter,* and *Cedecea* genera were increased in nasal swabs. In the pharynx, no genera were significantly enriched compared to nasal swabs. However, the families as Ruminococcaceae, Lachnospiraceae, and Bacteroidaceae were increased in the pharynx of HC subjects (Fig. 3J).

**Fig 4:**
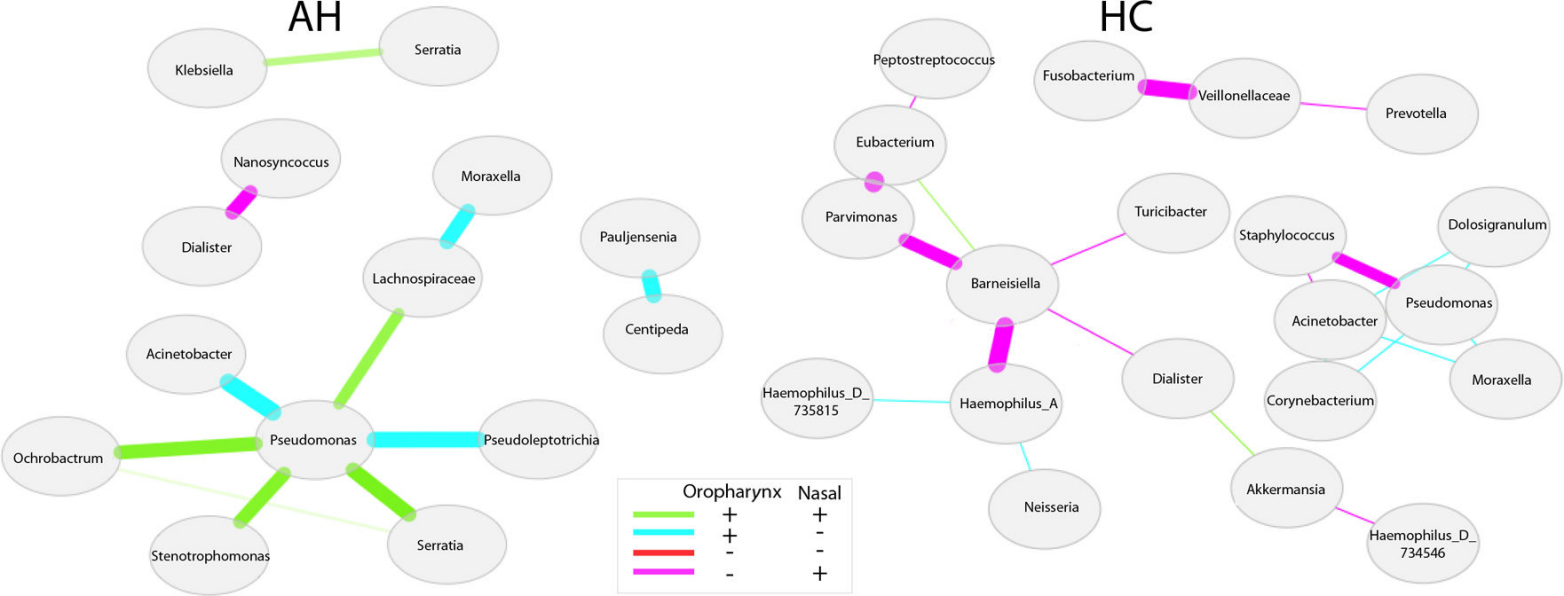
Differential network analysis. Pearson’s correlation test performed on the 80 most highly expressed genera in both pharyngeal and nasal microbiota, for AH and HC. The bacterial taxa resulted from the comparison of pharyngeal and nasal profiles by Fisher’s *z*-test (FDR corrected *P*-value ≥0.05). Nodes represent taxa, color edges identify connected components, and edge thickness indicates the rho absolute value.

The similarities between pharyngeal and nasal microbiota in AH were also evident by differential network analysis, which evidenced a lower number of edges in the AH network (*N* = 10) compared with the HC network (*N* = 19). Moreover, in AH, 5 out of 10 bacterial taxa were linked by positive correlations, while, in HC, 17 out of 19 bacterial taxa were linked by negative correlations (Fig. 4).

**Fig 5:**
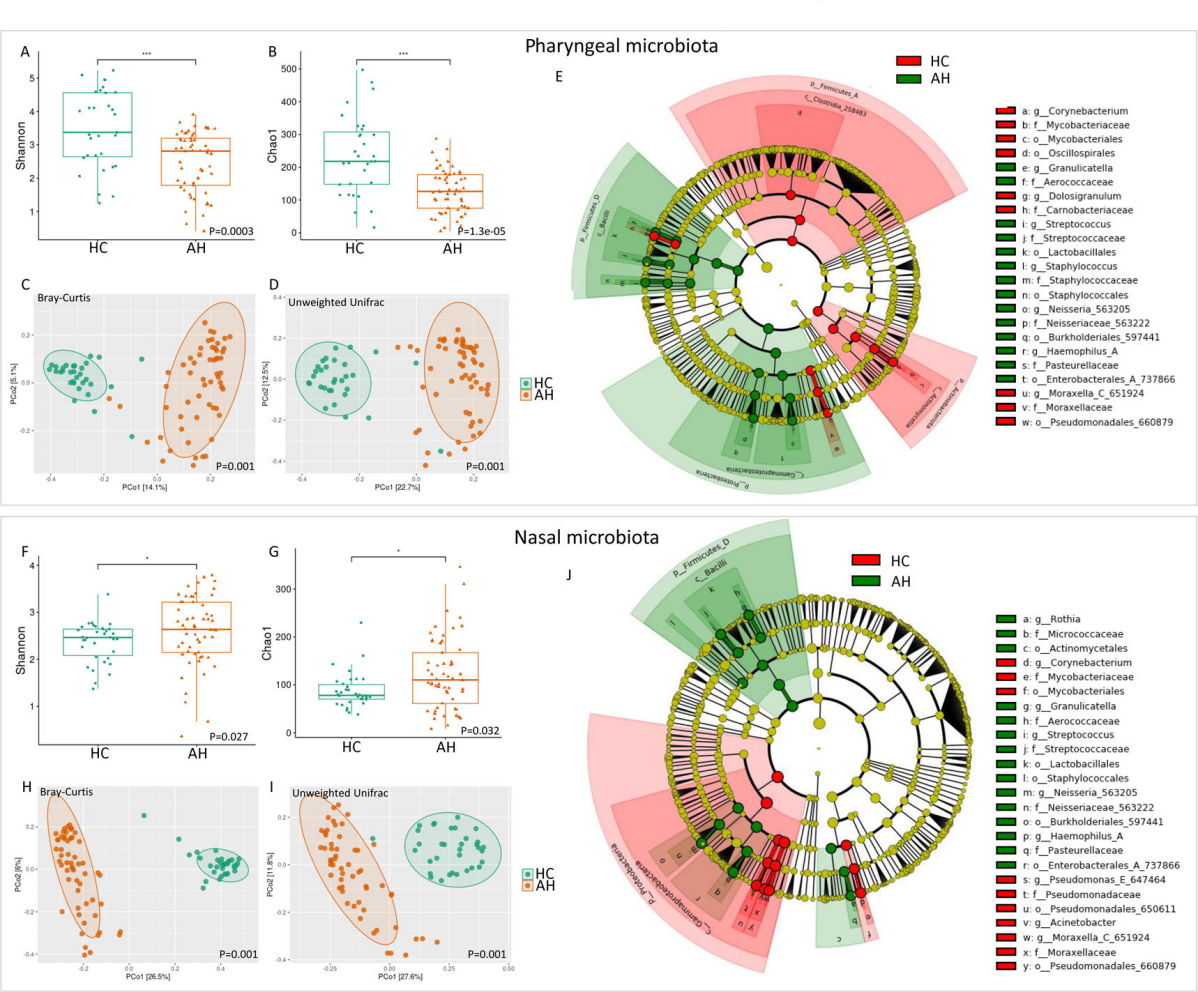
Pharyngeal (upper panel) and nasal (lower panel) microbial ecosystems of AH compared with HC. Alpha-diversity box plots of pharyngeal (**A and B**) and nasal (**F and G**) microbiota based on Shannon and Chao1 indexes. *P*-values obtained by the Mann–Whitney test are reported. Beta-diversity PCoA plots of pharyngeal (**C and D**) and nasal (**H and I**) microbiota, performed by Bray–Curtis and unweighted UniFrac algorithms. *P*-values obtained by the PERMANOVA test are reported. LEfSe cladograms of pharyngeal (**E**) and nasal (**J**) samples. Taxa and nodes highlighted in green and red were significantly more abundant in AH patients and HC, respectively.

We found a significant decrease in microbial richness in the pharynx of AH patients compared to HC (Fig. 5A and B). The differences in the pharyngeal ecosystem between AH and HC were also confirmed by β-diversity analysis, in which the separation between the two groups was evident (Fig. 5C and D). LEfSe analysis showed an increase of the genera *Granulicatella*, *Streptococcus*, *Staphylococcus*, *Neisseria*, and *Haemophilus*, as well as a reduction of *Corynebacterium*, *Dolosigranulum*, and *Moraxella* in pharyngeal swabs of AH patients (Fig. 5E).

Similarly, the nasal microbiota of the AH group showed a distinctive microbial fingerprint compared to HC (Fig. 5H and I). However, unlike the pharyngeal microbiota, this was characterized by an increase in microbial richness (Fig. 5F and G). Albeit with some differences, the nasal microbial markers were similar to those of the pharyngeal microbiota. In particular, we observed an increase of the genera *Rothia*, *Granulicatella*, *Streptococcus*, *Neisseria,* and *Haemophilus* and a reduction of *Corynebacterium*, *Pseudomonas, Acinetobacter,* and *Moraxella* in AH patients (Fig. 5J).

### The effects of probiotic administration on the pharyngeal and nasal microbiota

**Fig 6:**
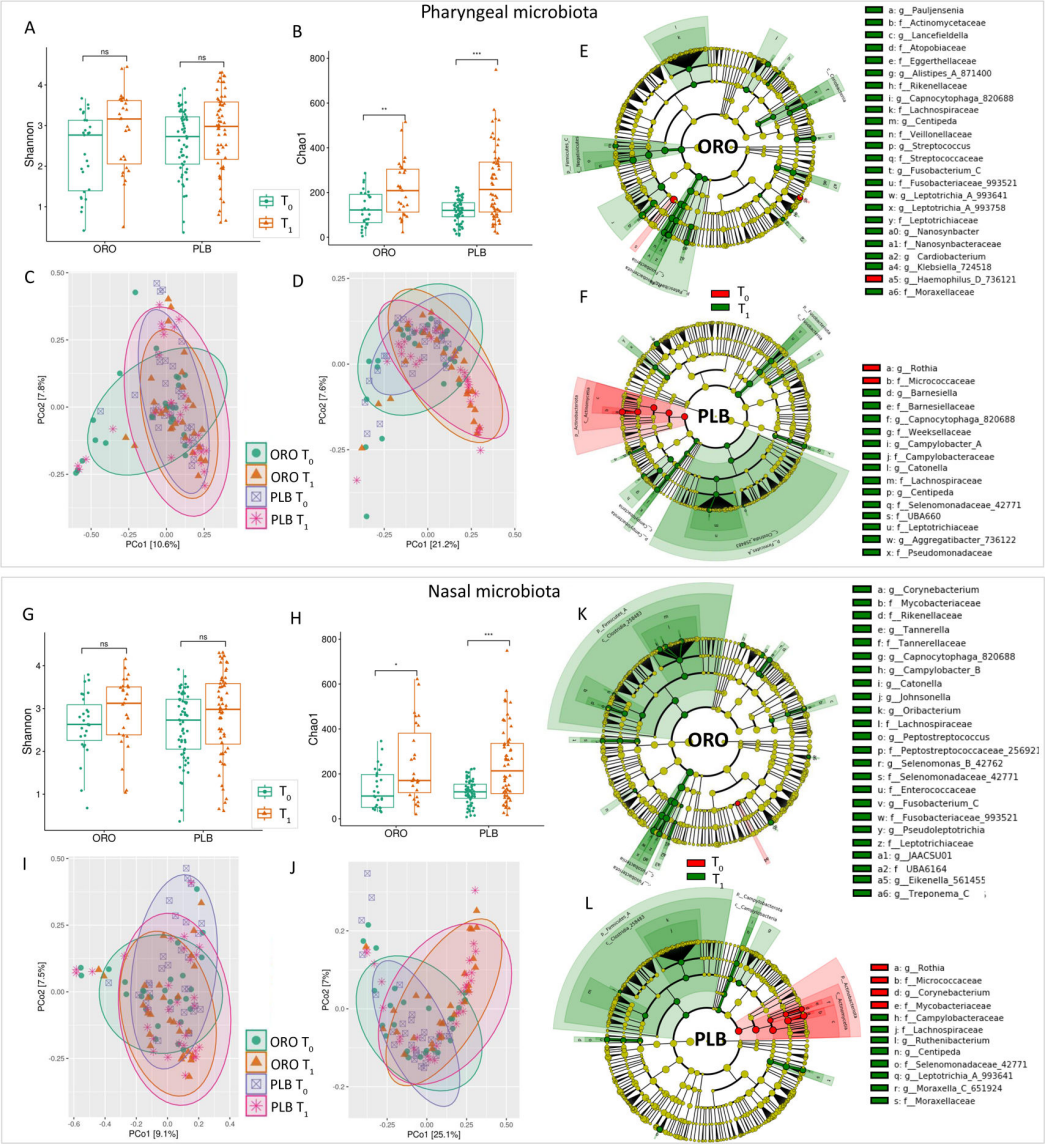
Probiotic effects on pharyngeal and nasal microbiota. Alpha-diversity box plots of pharyngeal (**A and B**) and nasal (**G and H**) microbiota based on Shannon and Chao1 indexes of probiotic-treated (ORO) and placebo (PLB) groups and before (***T*_0_**) and after (***T*_1_**) treatments. *P*-values obtained by the Mann–Whitney test are reported. Beta-diversity PCoA plots of pharyngeal (**C and D**) and nasal (**I and J**) microbiota, performed by Bray–Curtis and unweighted UniFrac algorithms of ORO and PLB groups and before (***T*_0_**) and after (***T*_1_**) treatment. The PERMANOVA test was applied to beta-diversity matrices. LEfSe cladograms of pharyngeal (**E and F**) and nasal (**K and L**) samples of ORO and PLB groups, respectively. Taxa and nodes highlighted in red and green were significantly more abundant before (***T*_0_**) and after (***T*_1_**) treatments, respectively.

We evaluated whether the administration of probiotics modified the microbial richness of the pharyngeal and nasal microbiota. We found that, following probiotic treatment, there was a significant increase of rare bacteria (Chao-1 index) in both the pharyngeal and nasal microbiota (Fig. 6A, B, G, and H). However, no significant differences in α-diversity were found when analyzing this data set with the Shannon index. Similarly, there were no significant differences in beta-diversity between treated and untreated patients (Fig. 6C, D, and G through J).

Next, we investigated the effects of probiotic treatment on the ecological composition of the bacterial populations in the pharynx and nasal passages. The analysis of the relative abundance of pharyngeal microbiota in the ORO group revealed an increase of *Pauljensenia*, *Lancefieldella*, *Alistipes*, *Capnocytophaga*, *Centipeda*, *Streptococcus*, *Fusobacterium*, *Leptotrichia*, *Nanosynbacter,* and *Klebsiella* and a reduction of *Haemophilus* after treatment (*T*_1_) (Fig. 6E). In the PLB group, we observed an increase of *Ruthenibacterium*, *Centipeda, Leptotrichia,* and *Moraxella*, as well as a reduction of *Rothia* at *T*_1_ (Fig. 6F). In the nasal microbiota, we found an increase of *Corynebacterium*, *Tannarella*, *Capnocytophaga*, *Campylobacter*, *Catonella*, *Johnsonella*, *Oribacterium*, *Peptostreptococcus*, *Selenomonas*, *Fusobacterium,* and *Pseudotrichia,* after ORO treatment (Fig. 6K). In the placebo group, we observed an increase of *Barnesiella*, *Capnocytophaga, Campylobacter, Catonella, Centipeda,* and *Aggregatibacter* and a reduction of *Rothia* and *Corynebacterium* at *T*_1_ (Fig. 6L).

**Fig 7:**
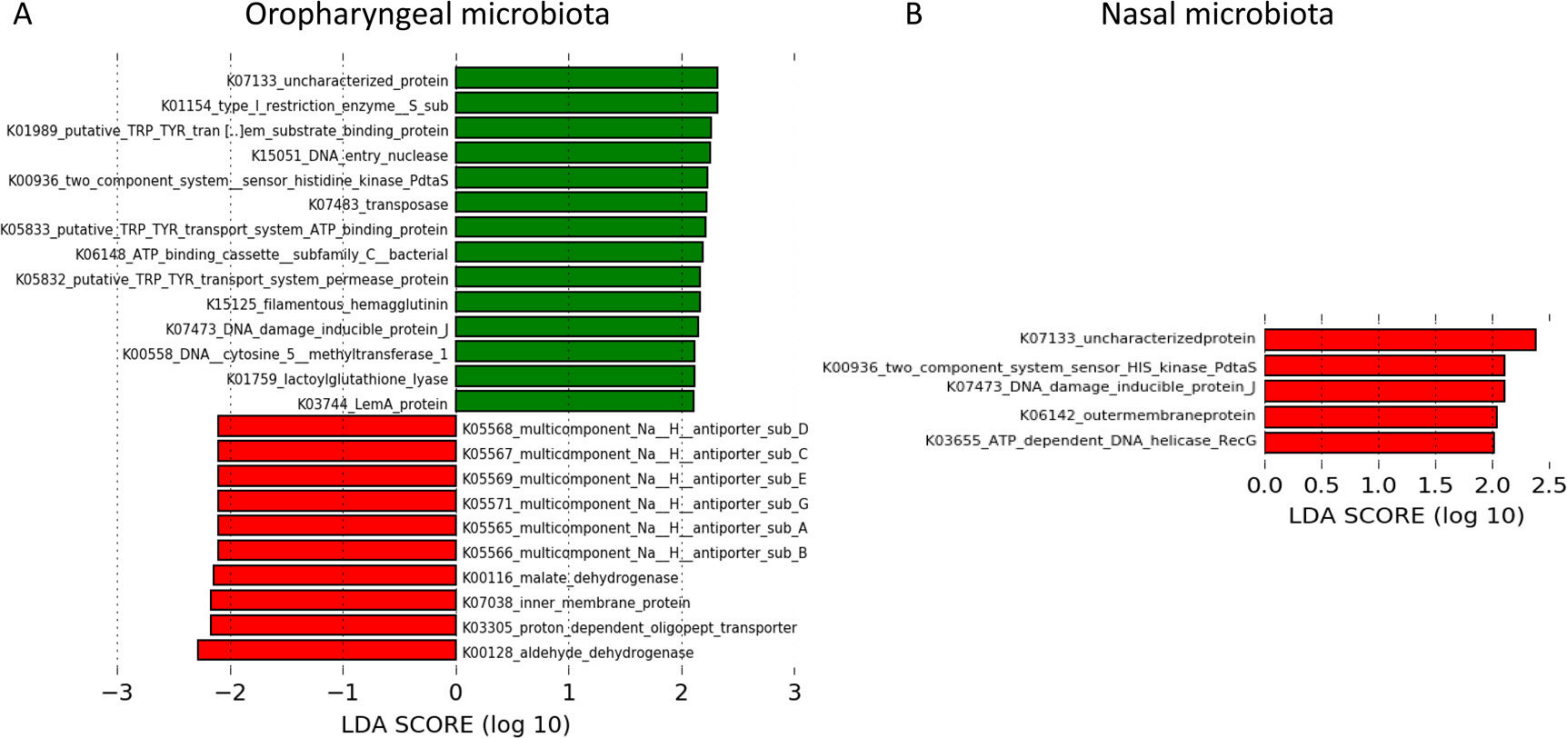
PICRUSt2 functional prediction. Predicted metabolic pathways statistically associated with before (***T*_0_**) and after (***T*_1_**) probiotic treatment in pharyngeal (**A**) and nasal (**B**) microbiota. Red bars represent pathways increased at *T*_0_; green bars represent pathways increased at *T*_1_.

To see whether these taxonomical shifts could also translate into changes in functional interactions between the microbiota and the host, we applied the PICRUSt algorithm to pharyngeal and nasal microbial populations before and after treatment. In the pharynx, we observed a marked decrease in signaling and cellular processes (k05565, k05566, k05567, k05568, k05569, k05571, and k03305), specifically involved in Na^+^/H^+^ transport, and of metabolism (k000116, k000128) after probiotic treatment. Moreover, we found an increase of KO involved in signaling and cellular processes (k01154, k01989, k06148, k05832, and k07473), metabolism (k00936, k00558, and k01759), and genetic information processing (k07483) and from uncharacterized pathways (k07133, k15051, and k03744) (Fig. 7A). In nasal samples, we observed an increase of signaling and cellular processes (k07473 and k06142), metabolism (k00936), and genetic information processing (k03655) pathways after treatment (Fig. 7B). In the pharynx of the placebo group, we observed an increase of the peptide/nickel transport system (k02032, k02033, and k02034), acyl CoA thioester hydrolase (k07101), chrA chromate transporter (k07240), HAE1 hydrophobic amphiphilic exporter (k03296), ArsR family transcriptional repressor (k03892), DNA damage-inducible protein J (k07473), and butanol dehydrogenase (k00100) and a reduction of bglA 6 phospho beta glucosidase (k01223) at *T*_1_ compared to *T*_0_ (Fig. S2A). In the nose of the placebo group, we only observed the reduction of one pathway, related to the HAE1 hydrophobic amphiphilic exporter (k03296) (Fig. S2B).

### Evaluation of probiotic effects on pharyngeal and nasal pathobionts with respect to AH grading

**Fig 8:**
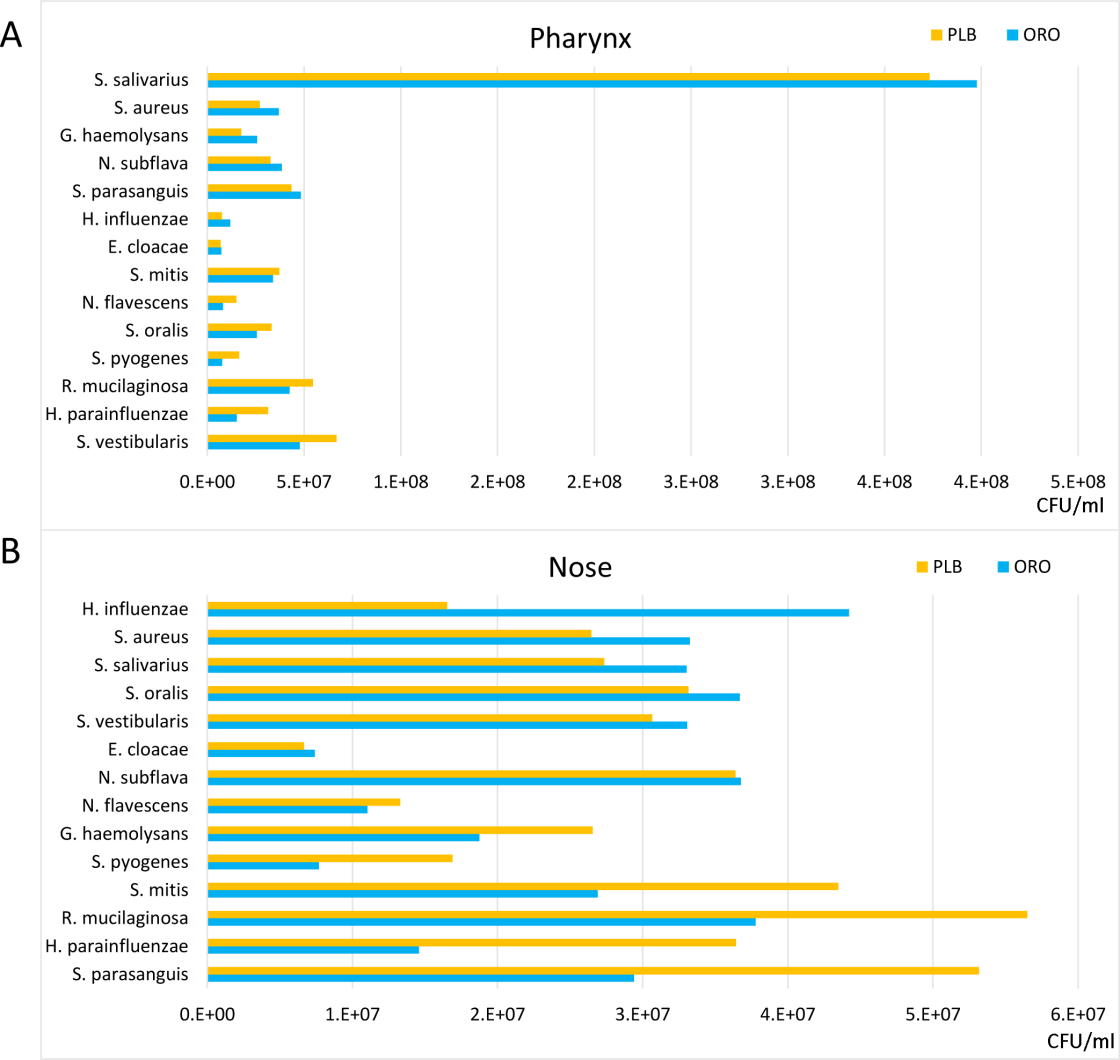
Treatment effects on pharyngeal and nasal pathobionts. Histograms of the pathobiont growth differences after treatment administration in the pharynx (**A**) and in the nose (**B**). In the horizontal axis are reported absolute values of growth differences between *T*_0_ and *T*_1_ in colony-forming units per milliliter.

We next investigated whether the presence of known pathobionts in the pharynx and nose of patients would be affected following probiotic treatment. Interestingly, we found an overall reduction of pathobionts following treatment with both the probiotic spray and the placebo (Table S4). However, probiotic treatment resulted in a greater growth reduction of *S. salivarius*, *S. aureus*, *G. haemolysans*, *N. subflava*, *S. parasanguis*, *H. influenzae*, and *E. cloacae* in the pharynx, and *H. influenzae*, *S. aureus*, *S. salivarius*, *S. oralis*, *S. vestibularis*, *E. cloacae*, and *N. subflava* in the nose (Fig. 8). These results suggest that the use of probiotics did have an inhibitory effect on the growth of some pathobionts, particularly on *H. influenzae*, which was also confirmed in our taxonomic analysis.

We next decided to stratify our patient cohort by AH grading, in order to evaluate the probiotic effects on pharynx and nose pathobionts with respect to AH severity.

**Fig 9:**
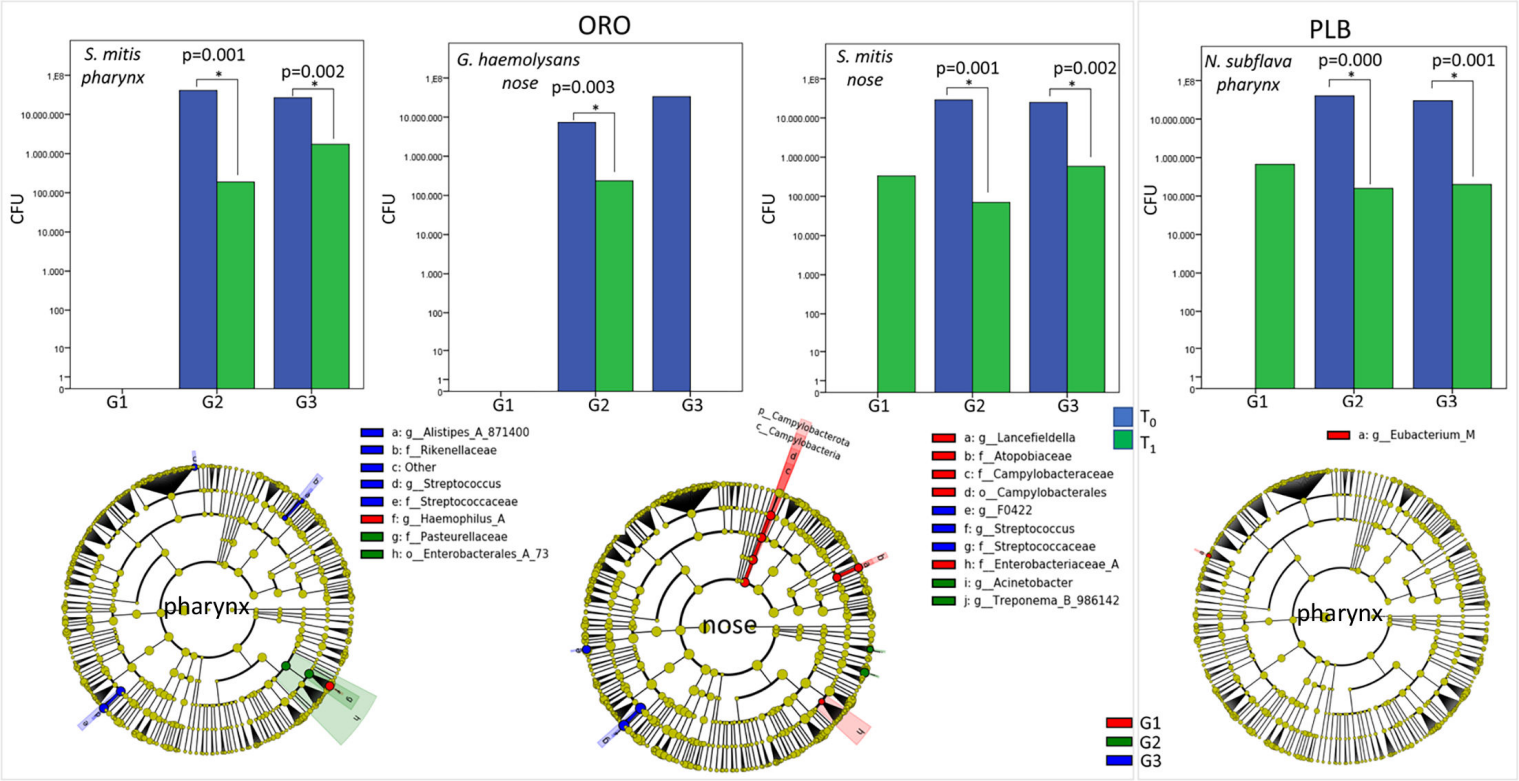
Treatment effects on pharyngeal and nasal bacteria in respect to AH grading. (Upper panel) Histograms of CFU of bacteria resulted in the statistically significant grouping of patients by AH grading. In *y*-axis, CFU; in *x*-axis, AH grading. *P*-values were calculated by the Wilcoxon test. (Lower panel) LEfSe cladograms of pharyngeal and nasal microbiota obtained by grouping patients on the basis of AH grading.

Interestingly, after probiotic treatment, we found a statistically significant decrease of *S. mitis* in the pharynx and nose of patients with G2 and G3 AH grading, and *G. haemolysans* in the nose of patients with G2 AH grading (Fig. 9). In the PLB group, *N. subflava* was significantly decreased in the pharynx of patients with G2 and G3 AH grading (Fig. 9).

Finally, we tested the effects of probiotic treatment on the pharyngeal and nasal microbiota with respect to AH grading. In the ORO group, LEfSe analysis revealed an increase of *Alistipes*, Rikenellaceae, *Streptococcus,* and Streptococcaceae in G3, Pasteurellaceae and Enterobacteriales in G2, and *Haemophilus* in G1 in the pharyngeal microbiota. In the nasal microbiota, we observed an increase of *Lancefildella*, Atopiobiaceae, Campylobacteriaceae, and Enterobacteriaceae in G1, *Streptococcus* and Streptococcaceae in G3, and *Acinetobacter* and *Treponema* (B_986142) in G2. In the PLB group, only *Eubacterium* was increased in the pharyngeal microbiota in G1 (Fig. 9).

## DISCUSSION

In this study, we investigated the pharyngeal and nasal microbiota in patients with AH compared with healthy children. Furthermore, we tested the effects of probiotic spray administration on disease symptoms and both microbiota profiles, to evaluate the possible use of this microbial therapy as an adjuvant therapy for AH patients.

To our knowledge, this is the first study that used this multiple exploratory approach, comparing two different microbial ecological niches and evaluating the microbial changes before and after oral probiotic treatment in children.

Recently, a meta-analysis reported that probiotics were more effective than the placebo in suppressing URTI, resulting in a reduced need for antibiotics, in both adults and children suffering from URTIs (25–27). Similarly, the use of a spray containing a mix of *S. salivarius* 24SMBc and *S. oralis* 89a in children significantly reduced the number of Group A beta-hemolytic streptococcus infectious episodes (28) and prevented recurrent acute otitis media (29) and URTI (30).

However, comparative evaluations of different probiotic formulations have shown that not all have the same biological activity but that each has specific peculiarities in terms of mechanism of action and efficacy (31). Moreover, the method of probiotic administration may also influence their efficacy (31). While most probiotics are ingested, an alternative mode of delivery is through a nasal or oral spray. The advantage of probiotic sprays could be due to their direct application, allowing for antagonistic interactions among microbes in the nasopharynx by changing the microenvironment and, thus, modulating pathobiont invasiveness (32). Moreover, orally administered nonencapsulated probiotics are easily destroyed by the harsh conditions within the human gastrointestinal tract, and only a small fraction of ingested probiotics are able to actually reach the large intestine (33), resulting in minimal effects on the taxonomic composition of the gut. On the other hand, the mechanism by which an oral probiotic spray exerts its effect on reducing URTI could be explained by direct *in situ* competition for the ecological niche between the commensal bacteria (probiotic) and pathobionts, decreasing the number of respiratory infections.

Our results suggest that the probiotic spray reduced the number of URTIs during the study period, consistent with other studies on respiratory infections in children (14). Probiotics can interact with other microbes of the human microbiome by the production of antimicrobials, competitive colonization, and inhibition of pathogen growth (e.g., by changing the pH in the niche) (34). Moreover, probiotics can have various immunomodulatory functions, including T helper cell 1 (Th1)/T helper cell 2 (Th2) immune balance restoration, stimulation of regulatory T cells, regulation of cytokines (35), and also modulation of mucosal IgA levels (36).

Generally, under physiological conditions in the upper respiratory tract, bacterial richness and evenness vary dramatically between niches, with the highest richness and evenness observed in the oropharynx and oral cavity (34). Contrastingly, the anterior nares harbor a bacterial microbiome exhibiting low biodiversity and are thereby more comparable to other areas covered by a skin-like epithelium (37–39). Our results in healthy children were consistent with these findings. However, in AH patients, the nasal microbiota richness seemed to be increased, more closely resembling that of the pharynx. Indeed, when compared to HC, we observed a decrease of α-diversity indices in the pharynx and an increase in the nose in AH patients. The differences in the AH microbiota profiles were also evident by β-diversity analyses, which highlighted an undefined sample cluster between the pharynx and the nose in AH, two distinct clusters between the pharynx and the nose in HC, and two separated clusters between AH and HC, in both sampling sites. The ratio between the pharyngeal and nasal microbial α-diversity index was significantly decreased in AH with respect to HC, suggesting an influence of AH on the decrease of microbiota richness. Moreover, a decrease in microbial diversity, richness, and evenness has been observed in chronic rhinosinusitis patients (40–42) and patients with respiratory diseases, including viral infections (43, 44).

Mechanistically, increased tissue inflammation in AH may change the physiological conditions of the nasal and pharyngeal ecological niches, thus favoring the overgrowth of certain bacterial genera or species. Indeed, despite the reported differences between the two, the pharyngeal and nasal microbiota of AH patients did share some microbial markers. This overlap in bacterial taxa could be explained by the proximity of the body sites and also the common source of drainage, i.e*.,* the nasopharynx, sinuses, and nasal cavity (38, 45, 46). On the other hand, variation between the two could be due to different bacterial colonization sources, such as the mouth and saliva, ingestion for the pharyngeal microbiota, and air filtering for the nasal one. Moreover, these two body sites show differences in the tissue surface (e.g., mucosal cell typology, immune system effectors, and mucus secretion), temperature, and pH, all of which can favor the colonization and growth of certain bacterial species over others (47). The increased inflammation in AH patients could further alter these physiological conditions in the nose and pharynx, leading to more evenness of microbial inhabitants between these two sites (45). In general, the AH network showed decreased connectivity with a low number of connected taxa. A bacterial network with lower connectivity may indicate more competition among taxa and has been previously associated with pathogenic states (48, 49).

Interestingly, among the differentially expressed taxa pre- and post-probiotic administration, we observed a reduction of *Haemophilus* in the pharyngeal microbiota of treated patients. *Haemophilus* is a typical inhabitant of the oral microbiota, but it could be also considered a potential pathobiont (34, 50). The probiotic effects on the nasal microbiota resulted in fewer changes than those observed in the pharynx. However, it is important to note that the nasal bacterial community constantly changes due to the openness of the nostrils to the air, varying its composition during the seasons, and in response to air pollution, host lifestyle, and other environmental factors (50–53). Therefore, all these potentially confounding factors should be considered in study cohort stratification. In our study, seasonality was only found to significantly influence the pharyngeal microbiota, showing the increase of *Akkermansia, Klebsiella*_724518, *Dialister, Neobacillus, Mannheimia, Clostridium*_AP, and *Eisenbergiella* in autumn, *Bulleidia* and *Catonella* in spring, and *Moraxella*_C_651924, *Avispirillum,* and *Aphodomorpha* in winter. Except for *Moraxella*_C_651924, which was also significantly affected in AH versus HC, the remaining bacterial taxa were exclusively correlated with seasonality.

Moreover, we found bacteria that inhabited both body sites and whose presence was not influenced by probiotic administration, such as *Capnocytophaga, Centipeda*, *Campylobacter,* and *Catonella*. In fact, these bacteria were present in both ORO and PLB groups after treatment. Conversely, *Corynebacterium* was increased in the nasal microbiota after probiotic supplementation and reduced in the PLB group. Finally, *Fusobacterium* was increased after probiotic treatment in both pharyngeal and nasal samples, and *Rothia* was decreased only in the PLB microbiota.

However, our results revealed that the effects of probiotics were more evident on the functional characteristics of the pharyngeal and nasal microbiota. Interestingly, in the pharyngeal microbiota, we observed a marked reduction of the multicomponent Na^+^/H^+^ antiporter subunits in AH, after the administration of the probiotic. These antiporters exert appropriate responses to environmental conditions, especially during stress conditions (54). We can speculate that probiotic treatment could reduce the environmental stress conditions present in AH. Moreover, probiotic administration seemed to enhance some beneficial metabolic pathways, such as prokaryotic defense systems, tryptophan/tyrosine transport system, ATP-binding cassette, and the two-component system. The ATP-binding cassette has a role in the import of essential nutrients and the export of toxic molecules (55). The prokaryotic defense and the two-component systems enable bacteria to adapt to various environmental changes and resist the colonization of invading pathogenic microbiomes (56, 57). Tyrosine and tryptophan are precursors of different neurotransmitters in the host (58). However, while there is a lot of literature on the role of the intestinal microbiota in the production and regulation of these neurotransmitters (59), there is a marked lack of studies on the potential role of extra-gut microbiota.

In the placebo group, we found an increase in pathways involved in stress resistance, which prevents microorganisms from the toxic effects of Ni^2+^ (60), resistance to chromate (61), and recovery from DNA damage (62). Though we found far fewer differences in the PLB group than we did in the ORO group, these results show that some significant changes do occur in the pharyngeal and nasal microbiota over time, despite the lack of administration of any active medication.

Finally, we evaluated whether probiotics could affect the growth of pathobionts selected among those attributable to the pathogenesis of AH. In fact, the upper airway microbiota is composed of opportunistic pathogens, which traverse the commensal–pathogen continuum (34, 38, 51). A reduction of all pathobionts in the microbiota of both sites was observed in both groups, due to the effect of the immune system acting on the adenotonsillar microbiota in immunocompetent children. However, the reduction in the growth of *S. salivarius*, *S. aureus*, *G. haemolysans*, *N. subflava*, *S. parasanguis*, *H. influenzae*, and *E. cloacae* in the pharynx and *H. influenzae*, *S. aureus*, *S. salivarius*, *S. oralis*, *S. vestibularis*, *E. cloacae*, and *N. subflava* in the nose was greater in the treated group than it was in the placebo group, which indicates that the probiotic spray helped to reduce the growth of these pathobionts. Moreover, the effect of probiotics in reducing the growth of *H. influenzae* was consistent with the metataxonomic results.

Moreover, the probiotic seemed to reduce the number of colonies of *S. mitis* in both the pharynx and the nose and *G. haemolysans* in the nose alone, in patients with a high grade of AH, indicating a positive correlation between probiotic effect and disease severity.

Although our results are novel and promising, this study has some limitations. First, the sample size was limited by the COVID-19 pandemic, which caused a restriction in subject enrolment. A larger cohort could have helped to overcome possible confounding effects, even if minimal, due to the influence of seasonality on the upper airway microbiota. Secondly, even though the microbiota profiling of the lymphoid tissue itself or the tonsillar crypts could be more informative, we carried out nose and pharynx swab sampling, to provide a less invasive sampling option in such young children and the absence of sedation. In conclusion, this study offers a comprehensive overview of the pharyngeal and nasal microbiota associated with health and AH, increasing the knowledge of the complex host–bacteria relationships in these specific and important body sites. Moreover, our results show that the probiotic had no clear adverse effects on the health of AH patients, conferred partial protection against URTIs, and reduced the presence of the pathobiont *Haemophilus*. Indeed, there were significantly more URTIs reported in the PLB group than in the ORO group, suggesting that probiotic administration did in fact have a partially protective effect against URTIs. Moreover, probiotic supplementation led to an increase of beneficial metabolic pathways in the pharyngeal microbiota, which were not observed in the placebo group. Finally, the probiotic reduced the concentration of the pathobionts *S. mitis* and *G. haemolysans* in relation to the severity of AH.

## Data Availability

All raw sequencing reads are available at the NCBI BioProject database (PRJNA1088074) (https://submit.ncbi.nlm.nih.gov/subs/sra/).

## SUPPLEMENTAL MATERIAL

Supplemental material spectrum.00728-24-s0001.docx Fig. S1 and S2; Tables S1 to S4.

ASM does not own the copyrights to Supplemental Material that may be linked to, or accessed through, an article. The authors have granted ASM a non-exclusive, world-wide license to publish the Supplemental Material files. Please contact the corresponding author directly for reuse.
