# Supplementary material for: Changes in the pharyngeal and nasal microbiota in pediatric patients with adenotonsillar hypertrophy
Source: Microbiol Spectr. 2024 Sep 9;12(10):e00728-24. doi: 10.1128/spectrum.00728-24 (PMC11449029; doi:10.1128/spectrum.00728-24)
Supplement: Supplemental material — Fig. S1 and S2; Tables S1 to S4. [file spectrum.00728-24-s0001.docx]

**Supplementary materials**

**
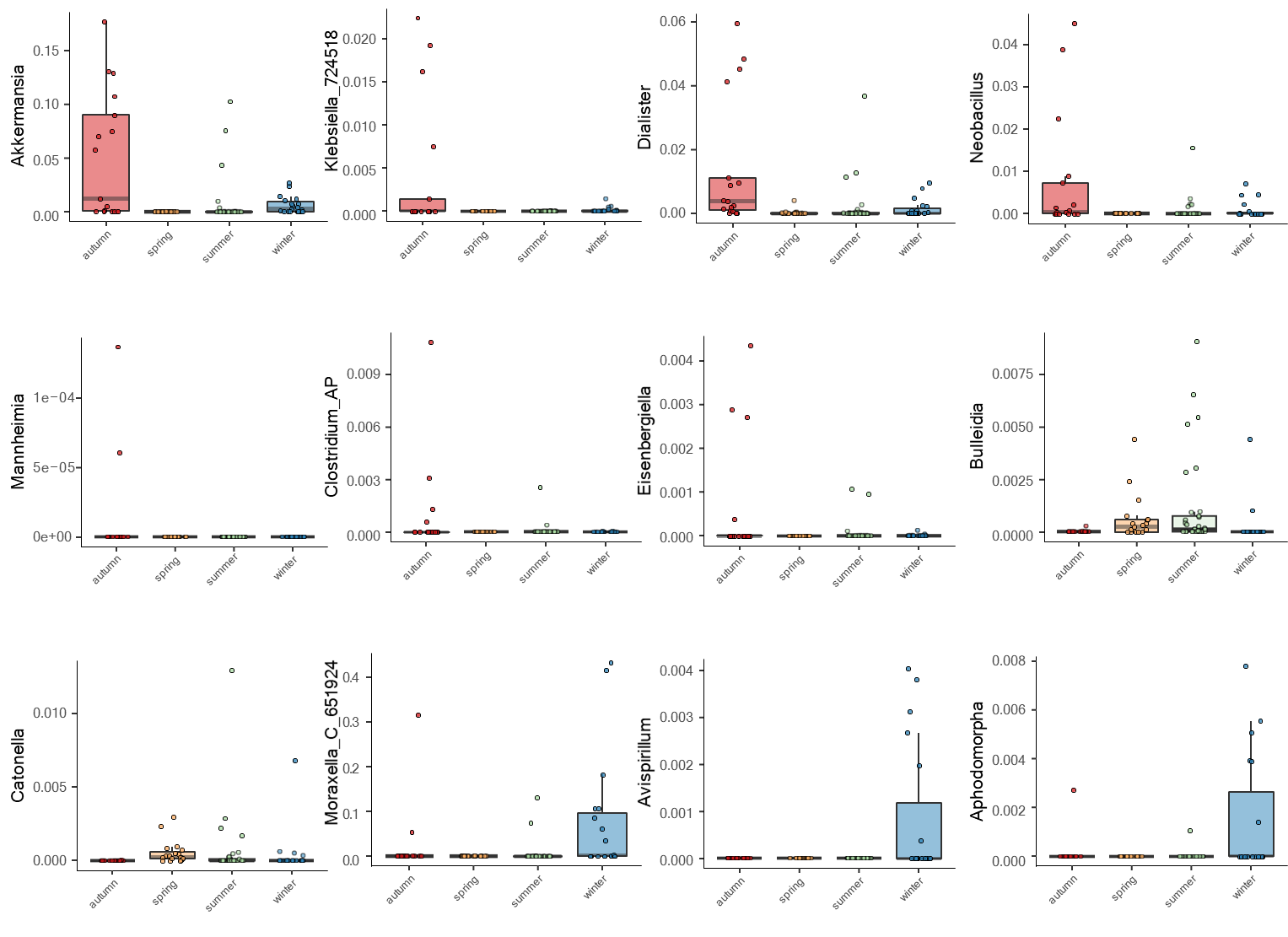
**

**Supplementary Figure S1. Box plots of microbial taxa influenced by seasons.** Bacterial taxa selected by p value FDR corrected ≤0.05 from Maaslin2 analysis.

**
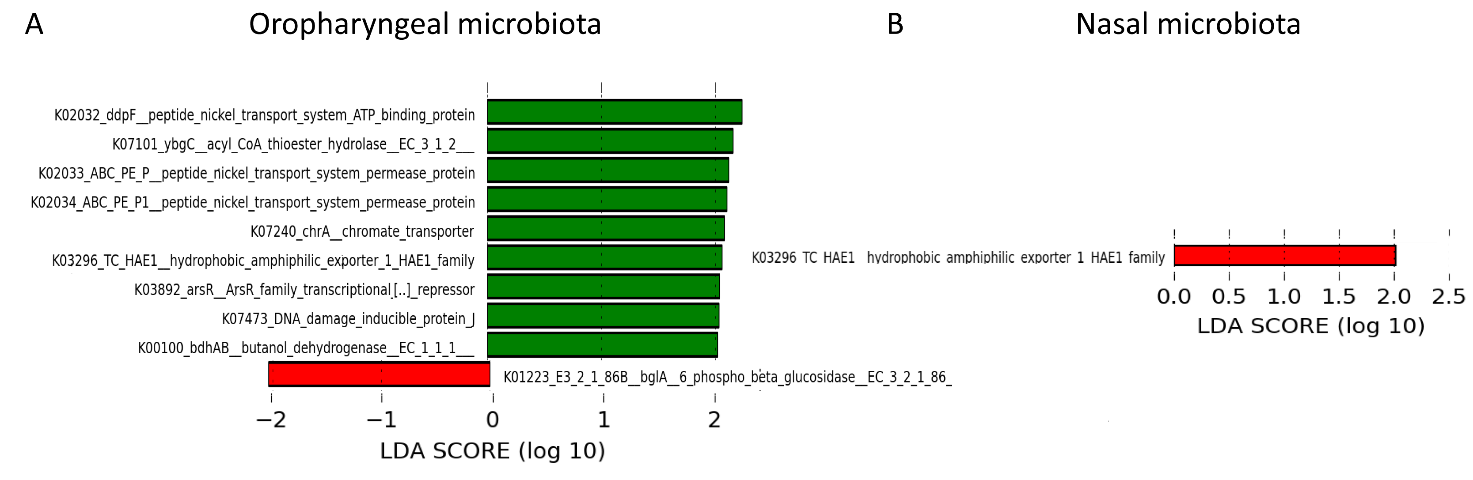
**

**Supplementary Figure S2. PICRUSt2 functional prediction.** Predicted metabolic pathways statistically associated to before (T_0_) and after placebo (T_1_) treatment in pharyngeal (**A**) and nasal (**B**) microbiota. Red bars represent pathways increased at T_0_; green bars represent pathways increased at T_1_. LDA, linear discriminant analysis.

**Supplementary Table S1.** Relative abundance of ASVs of pharyngeal and nasal microbiota (mean values amongst groups ≥ 0.1).

| Taxonomy | Pharyngeal microbiota | | | | | | Nasal microbiota | | | | | |
| --- | --- | --- | --- | --- | --- | --- | --- | --- | --- | --- | --- | --- |
| Phylum | HC | AH | ORO-T0 | ORO-T1 | PLB-T0 | PLB-T1 | HC | AH | ORO-T0 | ORO-T1 | PLB-T0 | PLB-T1 |
| Actinobacteriota | 15.77 | 6.09 | 3.86 | 6.26 | 8.09 | 3.75 | 11.98 | 7.34 | 4.56 | 5.54 | 9.85 | 4.00 |
| Bacteroidota | 13.79 | 7.84 | 7.52 | 8.80 | 8.14 | 12.13 | 0.38 | 5.45 | 4.67 | 9.66 | 6.15 | 10.63 |
| Campylobacterota | 0.17 | 0.31 | 0.30 | 0.60 | 0.31 | 0.82 | 0.00 | 0.24 | 0.28 | 0.70 | 0.20 | 0.56 |
| Firmicutes_A | 22.97 | 0.76 | 0.92 | 1.67 | 0.62 | 1.72 | 0.11 | 0.58 | 0.54 | 1.94 | 0.61 | 1.29 |
| Firmicutes_C | 2.17 | 4.76 | 3.16 | 7.76 | 6.21 | 8.90 | 0.12 | 4.46 | 4.07 | 6.11 | 4.81 | 8.35 |
| Firmicutes_D | 14.31 | 33.66 | 38.07 | 31.56 | 29.70 | 31.68 | 10.09 | 28.99 | 30.64 | 27.85 | 27.49 | 31.67 |
| Fusobacteriota | 3.06 | 4.04 | 5.64 | 9.21 | 2.60 | 8.57 | 0.08 | 3.38 | 4.42 | 11.70 | 2.45 | 5.20 |
| Methanobacteriota_A_1229 | 0.12 | 0.00 | 0.00 | 0.00 | 0.00 | 0.00 | 0.00 | 0.00 | 0.00 | 0.00 | 0.00 | 0.00 |
| Patescibacteria | 0.08 | 0.16 | 0.08 | 0.35 | 0.22 | 0.28 | 0.03 | 0.20 | 0.19 | 0.22 | 0.21 | 0.23 |
| Proteobacteria | 20.96 | 42.32 | 40.38 | 33.75 | 44.06 | 31.91 | 76.80 | 49.31 | 50.57 | 36.11 | 48.18 | 37.74 |
| Spirochaetota | 0.13 | 0.02 | 0.03 | 0.01 | 0.02 | 0.07 | 0.00 | 0.01 | 0.00 | 0.05 | 0.02 | 0.06 |
| unidentified | 2.38 | 0.02 | 0.02 | 0.04 | 0.03 | 0.15 | 0.30 | 0.04 | 0.04 | 0.05 | 0.03 | 0.26 |
| Verrucomicrobiota | 3.91 | 0.01 | 0.00 | 0.01 | 0.01 | 0.01 | 0.02 | 0.01 | 0.01 | 0.08 | 0.01 | 0.00 |
| Genus |  | | | | | | | | | | | |
| Achromobacter | 2.31 | 0.05 | 0.11 | 0.01 | 0.00 | 0.01 | 0.00 | 0.01 | 0.03 | 0.01 | 0.00 | 0.01 |
| Acinetobacter | 0.16 | 0.81 | 1.69 | 2.91 | 0.02 | 3.24 | 26.91 | 1.28 | 2.67 | 1.27 | 0.03 | 2.97 |
| Aeromonas | 0.04 | 3.23 | 0.68 | 0.00 | 5.52 | 0.00 | 0.00 | 2.58 | 0.11 | 0.00 | 4.81 | 0.00 |
| Aggregatibacter_736122 | 0.04 | 0.06 | 0.06 | 0.32 | 0.06 | 0.89 | 0.01 | 0.09 | 0.11 | 0.06 | 0.07 | 1.50 |
| Akkermansia | 3.91 | 0.01 | 0.00 | 0.01 | 0.01 | 0.01 | 0.02 | 0.01 | 0.01 | 0.08 | 0.01 | 0.00 |
| Alloprevotella | 0.53 | 1.33 | 1.58 | 0.79 | 1.11 | 1.80 | 0.07 | 0.88 | 0.49 | 0.92 | 1.24 | 1.55 |
| Anaeroglobus | 0.00 | 0.19 | 0.07 | 0.16 | 0.29 | 0.20 | 0.00 | 0.09 | 0.11 | 0.09 | 0.07 | 0.15 |
| Bacteroides_H | 3.73 | 0.01 | 0.01 | 0.01 | 0.02 | 0.01 | 0.01 | 0.01 | 0.02 | 0.02 | 0.01 | 0.01 |
| CAG-83 | 2.38 | 0.00 | 0.00 | 0.00 | 0.00 | 0.00 | 0.00 | 0.00 | 0.00 | 0.00 | 0.00 | 0.00 |
| Campylobacter_A | 0.16 | 0.30 | 0.30 | 0.58 | 0.31 | 0.81 | 0.00 | 0.24 | 0.28 | 0.58 | 0.20 | 0.55 |
| Capnocytophaga_820688 | 0.03 | 0.17 | 0.28 | 0.29 | 0.07 | 0.36 | 0.01 | 0.12 | 0.21 | 0.41 | 0.05 | 0.17 |
| Cedecea | 0.00 | 0.00 | 0.00 | 0.00 | 0.00 | 0.00 | 2.95 | 0.00 | 0.00 | 0.00 | 0.00 | 0.00 |
| Centipeda | 0.00 | 0.08 | 0.08 | 0.28 | 0.07 | 0.35 | 0.01 | 0.12 | 0.13 | 0.46 | 0.10 | 0.38 |
| Chryseobacterium_796614 | 0.00 | 0.00 | 0.00 | 0.24 | 0.01 | 0.75 | 0.00 | 0.00 | 0.00 | 0.18 | 0.00 | 0.82 |
| Corynebacterium | 13.88 | 0.06 | 0.02 | 0.06 | 0.10 | 0.05 | 11.78 | 0.13 | 0.01 | 0.09 | 0.25 | 0.04 |
| Dialister | 1.05 | 0.03 | 0.04 | 0.14 | 0.02 | 0.03 | 0.01 | 0.01 | 0.01 | 0.05 | 0.01 | 0.05 |
| Dolosigranulum | 5.97 | 0.00 | 0.00 | 0.01 | 0.00 | 0.00 | 4.64 | 0.00 | 0.00 | 0.01 | 0.00 | 0.01 |
| F0422 | 0.10 | 0.16 | 0.13 | 0.52 | 0.18 | 0.20 | 0.03 | 0.10 | 0.05 | 0.32 | 0.14 | 0.37 |
| Faecalibacterium | 3.36 | 0.01 | 0.01 | 0.01 | 0.00 | 0.01 | 0.00 | 0.01 | 0.01 | 0.01 | 0.01 | 0.01 |
| Faecousia | 1.29 | 0.00 | 0.00 | 0.00 | 0.00 | 0.00 | 0.00 | 0.00 | 0.00 | 0.00 | 0.00 | 0.00 |
| Fusobacterium_C | 2.94 | 3.07 | 4.47 | 6.81 | 1.80 | 6.63 | 0.06 | 2.67 | 3.65 | 8.33 | 1.79 | 3.73 |
| Gemella | 0.52 | 2.14 | 2.61 | 1.60 | 1.73 | 2.05 | 0.10 | 2.84 | 3.10 | 1.60 | 2.60 | 2.43 |
| Granulicatella | 0.21 | 8.89 | 8.10 | 7.07 | 9.61 | 6.46 | 0.07 | 8.34 | 6.79 | 5.03 | 9.74 | 6.15 |
| Haemophilus_A | 1.07 | 12.54 | 10.97 | 9.99 | 13.95 | 11.59 | 0.01 | 15.62 | 10.70 | 9.00 | 20.05 | 9.47 |
| Haemophilus_D_734546 | 3.81 | 2.48 | 1.33 | 3.63 | 3.51 | 0.69 | 3.00 | 4.47 | 5.29 | 7.06 | 3.72 | 2.36 |
| Haemophilus_D_735815 | 0.23 | 4.13 | 3.80 | 3.56 | 4.43 | 5.05 | 0.06 | 5.03 | 5.59 | 4.06 | 4.53 | 7.82 |
| Haemophilus_D_736121 | 0.09 | 0.63 | 0.86 | 0.27 | 0.42 | 1.09 | 0.01 | 1.23 | 1.78 | 1.21 | 0.74 | 0.51 |
| Lachnoanaerobaculum | 0.04 | 0.21 | 0.23 | 0.64 | 0.19 | 0.51 | 0.00 | 0.10 | 0.13 | 0.58 | 0.07 | 0.20 |
| Lactococcus_A_343306 | 0.00 | 0.01 | 0.00 | 0.00 | 0.03 | 3.07 | 0.00 | 0.01 | 0.00 | 0.00 | 0.01 | 0.00 |
| Lancefieldella | 0.02 | 0.07 | 0.02 | 0.23 | 0.11 | 0.26 | 0.00 | 0.06 | 0.06 | 0.15 | 0.06 | 0.26 |
| Leptotrichia_A_993641 | 0.05 | 0.16 | 0.18 | 0.99 | 0.14 | 0.56 | 0.01 | 0.22 | 0.25 | 0.57 | 0.18 | 0.54 |
| Leptotrichia_A_993758 | 0.02 | 0.77 | 0.92 | 1.29 | 0.63 | 1.29 | 0.01 | 0.44 | 0.50 | 2.45 | 0.39 | 0.83 |
| Moraxella_C_651731 | 3.84 | 0.00 | 0.00 | 0.01 | 0.00 | 0.00 | 0.04 | 0.00 | 0.00 | 0.00 | 0.00 | 0.03 |
| Moraxella_C_651924 | 6.48 | 0.01 | 0.00 | 0.01 | 0.02 | 0.01 | 15.02 | 0.91 | 1.69 | 0.79 | 0.21 | 1.12 |
| Nanosynbacter | 0.01 | 0.12 | 0.03 | 0.23 | 0.20 | 0.07 | 0.00 | 0.13 | 0.15 | 0.13 | 0.11 | 0.10 |
| Neisseria_563205 | 0.63 | 11.60 | 11.16 | 12.24 | 12.00 | 8.35 | 0.08 | 12.50 | 13.82 | 12.04 | 11.31 | 11.03 |
| Ochrobactrum_A_499024 | 0.01 | 0.21 | 0.45 | 0.00 | 0.00 | 0.00 | 0.00 | 0.25 | 0.54 | 0.00 | 0.00 | 0.00 |
| Oribacterium | 0.01 | 0.14 | 0.18 | 0.28 | 0.10 | 0.23 | 0.00 | 0.08 | 0.10 | 0.27 | 0.06 | 0.24 |
| Pauljensenia | 0.06 | 0.21 | 0.15 | 0.40 | 0.26 | 0.25 | 0.03 | 0.30 | 0.24 | 0.48 | 0.36 | 0.23 |
| Phocaeicola_A_858004 | 4.52 | 0.01 | 0.01 | 0.01 | 0.01 | 0.01 | 0.01 | 0.01 | 0.01 | 0.01 | 0.01 | 0.00 |
| Porphyromonas_A_859423 | 0.15 | 0.48 | 0.55 | 1.42 | 0.42 | 1.14 | 0.05 | 0.70 | 0.36 | 0.99 | 1.00 | 1.09 |
| Porphyromonas_A_859424 | 1.14 | 0.15 | 0.25 | 0.03 | 0.06 | 0.28 | 0.00 | 0.01 | 0.02 | 0.12 | 0.00 | 0.16 |
| Prevotella | 1.00 | 5.38 | 4.51 | 5.61 | 6.17 | 7.07 | 0.06 | 3.57 | 3.37 | 6.59 | 3.74 | 6.41 |
| Pseudomonas_E_647464 | 0.11 | 0.08 | 0.16 | 0.00 | 0.00 | 0.31 | 27.09 | 0.02 | 0.05 | 0.00 | 0.00 | 0.20 |
| Rothia | 0.13 | 5.71 | 3.64 | 5.50 | 7.57 | 3.12 | 0.07 | 6.81 | 4.23 | 4.76 | 9.13 | 3.42 |
| Ruminococcus_E | 1.83 | 0.00 | 0.00 | 0.00 | 0.00 | 0.00 | 0.00 | 0.00 | 0.00 | 0.00 | 0.00 | 0.00 |
| Serratia_D_727245 | 0.00 | 1.77 | 3.69 | 0.00 | 0.05 | 0.00 | 0.25 | 0.01 | 0.00 | 0.00 | 0.02 | 0.00 |
| Serratia_D_727363 | 0.04 | 1.02 | 2.14 | 0.00 | 0.00 | 0.00 | 0.00 | 0.83 | 1.76 | 0.00 | 0.00 | 0.00 |
| Staphylococcus | 2.50 | 11.11 | 19.78 | 2.11 | 3.30 | 10.02 | 3.10 | 7.71 | 11.76 | 6.45 | 4.06 | 9.27 |
| Stenotrophomonas_A_615274 | 0.13 | 0.24 | 0.51 | 0.05 | 0.00 | 0.01 | 0.00 | 0.37 | 0.78 | 0.03 | 0.00 | 0.01 |
| Streptococcus | 3.44 | 10.98 | 7.25 | 20.55 | 14.33 | 9.93 | 2.14 | 9.93 | 8.79 | 14.53 | 10.95 | 13.63 |
| Veillonella_A | 0.41 | 4.17 | 2.55 | 6.62 | 5.63 | 7.99 | 0.08 | 4.13 | 3.74 | 5.04 | 4.48 | 7.31 |

**Supplementary Table S2.** Confounding factor analysis results obtained by microbiomeMarker v1.6.0 R package

|  | Confounder | Pseudo_F | p value |
| --- | --- | --- | --- |
| **Nasal microbiota AH vs HC** | Season | 1.18 | 0.24 |
| **Pharyngeal microbiota AH vs HC** | Season | 2.24 | 0.001 |

**Supplementary Table S3.** Bacterial taxa influenced by seasonality obtained by Microbiome Multivariable Association with Linear Model 2 (MaAsLin2) algorithm and selected for p value FDR corrected ≤0.005.

| **Feature** | **pval** | **FDR p value** |
| --- | --- | --- |
| ***Akkermansia*** | 0.000 | 0.005 |
| ***Catonella*** | 0.000 | 0.005 |
| ***Bulleidia*** | 0.002 | 0.037 |
| ***Dialister*** | 0.000 | 0.008 |
| ***Klebsiella_724518*** | 0.001 | 0.014 |
| ***Mannheimia*** | 0.003 | 0.046 |
| ***Aphodomorpha*** | 0.002 | 0.038 |
| ***Avispirillum*** | 0.000 | 0.005 |
| ***Clostridium_AP*** | 0.002 | 0.035 |
| ***Eisenbergiella*** | 0.003 | 0.044 |
| ***Moraxella_C_651924*** | 0.002 | 0.038 |
| ***Neobacillus*** | 0.000 | 0.006 |

**Supplementary Table S4.** Abundances (UFC/ml) of selected pathobionts cultured by pharyngeal and nasal swabs and compared before (T_0_) and after (T_1_) treatments tested by Wilcoxon’s test (p values ≤ 0.05).

|  |  | ORO group | | | | | PLB group | | | | |
| --- | --- | --- | --- | --- | --- | --- | --- | --- | --- | --- | --- |
|  |  | T_0_ | | T_1_ | |  | T_0_ | | T_1_ | |  |
|  |  | average | SD | average | SD | P value | average | SD | average | SD | P value |
| Pharyngeal swabs | *E. cloacae* | 7x10^6^ | 3x10^7^ | 4x10^4^ | 2x10^5^ | 0.157 | 7x10^6^ | 3x10^7^ | 3x10^4^ | 2x10^5^ | 0.137 |
|  | ***G. haemolysans*** | 3x10^7^ | 4x10^7^ | 2x10^5^ | 4x10^5^ | **0.004** | 2x10^7^ | 4x10^7^ | 8x10^4^ | 3x10^5^ | **0.013** |
|  | *H. influenzae* | 1x10^7^ | 3x10^7^ | 8x10^4^ | 3x10^5^ | 0.058 | 8x10^6^ | 3x10^7^ | 1x10^5^ | 3x10^5^ | 0.107 |
|  | ***H. parainfluenzae*** | 2x10^7^ | 4x10^7^ | 3x10^5^ | 4x10^5^ | **0.032** | 3x10^7^ | 5x10^7^ | 2x10^5^ | 4x10^5^ | **0.000** |
|  | *N. flavescens* | 8x10^6^ | 3x10^7^ | 5x10^2^ | 2x10^3^ | 0.115 | 2x10^7^ | 3x10^7^ | 4x10^3^ | 2x10^4^ | 0.019 |
|  | ***N. subflava*** | 4x10^7^ | 5x10^7^ | 2x10^5^ | 4x10^5^ | **0.000** | 3x10^7^ | 4x10^7^ | 2x10^5^ | 4x10^5^ | **0.000** |
|  | ***R. mucilaginosa*** | 4x10^7^ | 5x10^7^ | 2x10^5^ | 4x10^5^ | **0.000** | 5x10^7^ | 3x10^7^ | 2x10^5^ | 2x10^5^ | **0.000** |
|  | ***S. aureus*** | 4x10^7^ | 5x10^7^ | 1x10^5^ | 3x10^5^ | **0.000** | 3x10^7^ | 4x10^7^ | 2x10^5^ | 4x10^5^ | **0.001** |
|  | ***S. mitis*** | 3x10^7^ | 5x10^7^ | 6x10^5^ | 2x10^6^ | **0.000** | 4x10^7^ | 5x10^7^ | 1x10^5^ | 3x10^5^ | **0.000** |
|  | ***S. oralis*** | 3x10^7^ | 4x10^7^ | 3x10^5^ | 5x10^5^ | **0.004** | 3x10^7^ | 5x10^7^ | 5x10^4^ | 2x10^5^ | **0.000** |
|  | ***S. parasanguis*** | 5x10^7^ | 5x10^7^ | 2x10^5^ | 4x10^5^ | **0.000** | 4x10^7^ | 5x10^7^ | 2x10^5^ | 4x10^5^ | **0.000** |
|  | *S. pyogenes* | 8x10^6^ | 3x10^7^ | 8x10^4^ | 3x10^5^ | 0.139 | 2x10^7^ | 4x10^7^ | 2x10^5^ | 4x10^5^ | **0.020** |
|  | *S. salivarius* | 4x10^8^ | 2x10^9^ | 6x10^5^ | 5x10^5^ | 0.287 | 4x10^8^ | 2x10^9^ | 4x10^5^ | 5x10^5^ | 0.265 |
|  | ***S. vestibularis*** | 5x10^7^ | 5x10^7^ | 3x10^5^ | 5x10^5^ | **0.000** | 7x10^7^ | 2x10^8^ | 2x10^5^ | 4x10^5^ | 0.050 |
| Nasal swabs | ***E. cloacae*** | 7x10^6^ | 3x10^7^ | 0.000 | 0.000 | **0.000** | 7x10^6^ | 3x10^7^ | 0.000 | 0.000 | **0.00** |
|  | ***G. haemolysans*** | 2x10^7^ | 4x10^7^ | 1x10^5^ | 4x10^5^ | **0.017** | 3x10^7^ | 4x10^7^ | 1x10^5^ | 3x10^5^ | **0.002** |
|  | ***H. influenzae*** | 4x10^7^ | 5x10^7^ | 2x10^5^ | 4x10^5^ | **0.000** | 2x10^7^ | 4x10^7^ | 1x10^5^ | 3x10^5^ | **0.020** |
|  | ***H. parainfluenzae*** | 1x10^7^ | 4x10^7^ | 3x10^5^ | 4x10^5^ | **0.041** | 4x10^7^ | 5x10^7^ | 2x10^5^ | 4x10^5^ | **0.000** |
|  | *N. flavescens* | 1x10^7^ | 8x10^6^ | 7x10^4^ | 3x10^5^ | 0.079 | 1x10^7^ | 3x10^7^ | 3x10^4^ | 2x10^4^ | **0.039** |
|  | ***N. subflava*** | 4x10^7^ | 5x10^7^ | 3x10^5^ | 4x10^5^ | **0.000** | 4x10^7^ | 5x10^7^ | 3x10^5^ | 4x10^5^ | **0.000** |
|  | ***R. mucilaginosa*** | 4x10^7^ | 3x10^7^ | 4x10^5^ | 3x10^5^ | **0.000** | 6x10^7^ | 5x10^7^ | 2x10^5^ | 4x10^5^ | **0.000** |
|  | ***S. aureus*** | 3x10^7^ | 5x10^7^ | 7x10^4^ | 3x10^5^ | **0.001** | 3x10^7^ | 4x10^7^ | 2x10^5^ | 4x10^5^ | **0.002** |
|  | ***S. mitis*** | 3x10^7^ | 4x10^7^ | 2x10^5^ | 4x10^5^ | **0.003** | 4x10^7^ | 5x10^7^ | 3x10^5^ | 4x10^5^ | **0.000** |
|  | ***S. oralis*** | 4x10^7^ | 5x10^7^ | 3x10^5^ | 5x10^5^ | **0.000** | 3x10^7^ | 5x10^7^ | 2x10^5^ | 4x10^5^ | **0.000** |
|  | *S. parasanguis* | 3x10^7^ | 5x10^7^ | 2x10^5^ | 4x10^5^ | **0.002** | 5x10^7^ | 5x10^7^ | 2x10^5^ | 4x10^5^ | **0.000** |
|  | *S. pyogenes* | 8x10^6^ | 3x10^7^ | 1x10^5^ | 3x10^5^ | 0.139 | 2x10^7^ | 4x10^7^ | 1x10^5^ | 3x10^5^ | **0.017** |
|  | ***S. salivarius*** | 3x10^7^ | 5x10^7^ | 3x10^5^ | 5x10^5^ | **0.001** | 3x10^7^ | 4x10^7^ | 3x10^5^ | 5x10^5^ | **0.001** |
|  | ***S. vestibularis*** | 3x10^7^ | 5x10^7^ | 3x10^5^ | 4x10^5^ | **0.001** | 3x10^7^ | 5x10^7^ | 4x10^5^ | 5x10^5^ | **0.001** |
